# Predictors of Return to Driving in Patients With Acquired Brain Injury: A Systematic Review With Meta‐Analysis

**DOI:** 10.1155/oti/9280995

**Published:** 2026-06-09

**Authors:** Sujin Hwang, Chiang-soon Song

**Affiliations:** ^1^ Department of Physical Therapy, Division of Health Science, Baekseok University, Cheonan, Republic of Korea, bu.ac.kr; ^2^ Department of Occupational Therapy, College of Natural Science and Public Health and Safety, Chosun University, Gwangju, Republic of Korea, chosun.ac.kr

**Keywords:** automobile driving, brain injury, predictors, rehabilitation

## Abstract

**Objectives:**

This review aimed to comprehensively synthesise evidence on the therapeutic efficacy of driving rehabilitation for patients with acquired brain injury (ABI) and identify and analyse key predictors of successful return‐to‐driving.

**Methods:**

A comprehensive literature search was conducted using four databases: PubMed, Embase, Web of Science and ProQuest. Eleven studies (four randomised controlled trials and seven cross‐sectional studies) were included in the final analysis. Reporting completeness and methodological quality were assessed using PRISMA checklists. A random‐effects model was used to calculate the standardised mean differences (SMDs) and odds ratios (ORs).

**Main Findings:**

A qualitative analysis of cross‐sectional studies identified cognitive processing speed, reaction time and functional independence as frequently reported predictors of returning to driving, with a pooled meta‐analysis further highlighting lower initial injury severity (higher GCS scores) as a significant predictor of returning to driving. The meta‐analysis revealed that simulator‐ and VR‐based training significantly reduced driving errors (SMD = −1.19, 95% confidence interval [CI]: −1.75 to −0.64; *p* < 0.0001). The intervention significantly increased the likelihood of passing the on‐road driving test by approximately 3.37 times (OR = 3.37, 95% CI: 1.36–8.35).

**Conclusion:**

The results of this review suggest that simulator‐based interventions may positively impact driving performance and current cognitive and functional activities, as well as psychological self‐efficacy, and facilitat a successful return to driving in patients with ABI. These findings provide a baseline for developing clinical guidelines for the safe resumption of driving.

## 1. Introduction

In human life, driving is generally a daily activity even for adults with acquired brain injury (ABI) [1]. Previous studies have indicated that between 40% and 80% of patients with ABI and stroke resume driving [2, 3]. Restoring driving abilities is a functional goal of participation in intensive rehabilitation programmes [4]. The resumption of driving after ABI is influenced by complex factors, because patients with ABI may experience a range of physical, cognitive and visual impairments (e.g., visual field loss, diplopia and reduced acuity), which can lead to permanent disabilities and psychological issues, all of which can compromise safe driving [5, 6]. Driving function is a critical determinant of community reintegration in patients with ABI, making driving rehabilitation an essential component of post‐injury intensive rehabilitation programmes [7–10].

In rehabilitation settings, driving rehabilitation after ABI often receives insufficient attention, with a predominant focus on the recovery of activities of daily living and functional mobility [6]. Ensuring a safe return to driving involves possessing vital cognitive skills, such as a high speed of cognitive processing, capacity for both sustained and divided attention, rapid reaction times to manage sudden dangerous situations and situational awareness to accurately assess complex road environments. Therefore, a delicate balance between patient safety and autonomy is required to ensure optimal outcomes. Several countries have implemented policies that require healthcare professionals to identify individuals whose medical conditions may pose a risk to safe driving [11, 12].

Although previous research has explored the rates and predictors of driving resumption after ABI, evidence regarding the effectiveness of specific interventions, particularly emerging technologies such as driving simulators and VR, remains fragmented, posing a challenge for developing evidence‐based clinical guidelines [13, 14]. There is also a lack of consensus regarding whether traditional clinical assessments or functional activity measures are better predictors of safe driving during the recovery period after ABI [3, 14, 15]. Therefore, this systematic review and meta‐analysis aimed to achieve two primary objectives: (1) to identify and evaluate the key predictors of returning to driving following ABI and (2) to evaluate the therapeutic efficacy of driving‐based rehabilitation (e.g., simulators, VR and on‐road) compared to standard care or nondriving rehabilitation on driving resumption.

## 2. Methods

This study was a systematic review with meta‐analysis. The review protocol was registered with an international systematic review registry, PROSPERO (registration no. CRD42024513814). Two authors independently performed all stages of the literature search for articles on driving rehabilitation in patients with an ABI. Records lacking specific outcome measures, means, standard deviations or sample sizes necessary for calculating standard errors and effect estimates for the meta‐analysis were excluded. This was crucial for robust meta‐analytic calculations using a random‐effects model.

### 2.1. Eligibility Criteria

The inclusion criteria for the studies were as follows: (1) individuals with ABI without other neurological disorders (aged ≥ 18 years), (2) research articles on automobile driving rehabilitation or studies on predictors of return to driving after ABI, (3) studies written only in English, (4) studies in humans and (5) studies reporting the full text. The exclusion criteria for the study were as follows: (1) nonhuman studies; (2) not original articles; (3) grey literature, such as theses, conference or academic papers and abstracts; and (4) patients who started driving after the onset of ABI.

### 2.2. Information Sources and Search Strategy

A literature search was conducted using PubMed and three other academic databases (Embase, Web of Science and ProQuest) until 31 October 2025. The search strategy was a combination of the following Medical Subject Headings (MeSH) and related terms: (brain injur∗ OR acquired brain injur∗ OR acute brain injur∗ OR brain damage) AND (driving OR automobile driving OR motor driving) AND rehabilitation. The full list of retrieved articles was analysed for randomised controlled trials (RCTs) evaluating the effectiveness of driving rehabilitation approaches for returning to driving in patients with ABI and cross‐sectional studies investigating variables predicting return to driving in patients with ABI.

### 2.3. Study Selection Process

After searching for studies on driving rehabilitation for patients with ABI across the four databases and summing the retrieved studies, we removed duplicate records from the title list of the selected studies. We also assessed the population, intervention, comparison and outcomes with study design (PICO‐SD) to investigate the effects of driving rehabilitation on driving skills and resumption of driving in patients with ABI. To evaluate research‐based evidence on the predictors of return to driving, a review was performed on the population, aim, confounding variables, measurement of intervention/exposure, outcome measures, statistical values and reported results.

### 2.4. Data Collection Process

A quantitative synthesis and qualitative relevance analysis were conducted on the driving rehabilitation of patients with ABI based on the studies collected. To analyse the risk of bias (RoB) in the RCTs, we collected data on random sequence generation, allocation concealment, patient blinding, outcome assessment blinding, incomplete outcome data and selective reporting from the selected studies. We also calculated the mean, standard deviation and number of participants for each of the outcome measures.

### 2.5. Quality Assessment and Reporting Adherence for the Included Cross‐Sectional Studies

Two distinct tools were used to comprehensively evaluate the quality of the included cross‐sectional studies. First, the Strengthening the Reporting of Observational Studies in Epidemiology (STROBE) checklist was used to assess the reporting quality and transparency [16]. Second, the Joanna Briggs Institute (JBI) critical appraisal checklist for analytical observational studies was used to assess the methodological quality and RoB [17].

### 2.6. Quality Assessment and Reporting Adherence for the Included RCT Studies

Both reporting quality and methodological validity were assessed to evaluate the quality of the four included RCTs. The reporting quality was assessed using the Consolidated Standards of Reporting Trials (CONSORT) 2010 checklist [18]. To quantitatively compare reporting fidelity across studies, this review developed a scoring system based on 25 essential items. Each item was assigned a score of 1 if adequately reported and 0 if not reported or insufficiently reported, resulting in a maximum possible total score of 25 for each study. The RoB was assessed using the Cochrane RoB tool to identify potential threats to internal validity using Review Manager (RevMan), version 5.4 [19].

### 2.7. Quantitative Synthesis of the Selected Studies

Effect sizes were determined based on data type; mean difference (MD) or standardised MD (SMD) was used for continuous variables, whereas odds ratios (ORs) were calculated for dichotomous outcomes. All estimates were reported with 95% confidence intervals (CIs) [19]. To evaluate the consistency across the included studies, statistical heterogeneity was assessed using Higgins *I* ^2^ statistics. Heterogeneity levels were categorised as low (*I* ^2^ of 25%–50%), moderate (*I* ^2^ of 50%–75%) or high (*I* ^2^ > 75%) [19]. Quantitative synthesis was performed when two or more studies reported comparable outcomes with sufficient statistical data. Random‐effects models were used to account for potential clinical and methodological heterogeneity among studies. Quantitative pooling was not performed for outcomes reported in only one study. The individual study effect estimate and its 95% CI were presented descriptively to avoid interpreting single‐study findings as pooled meta‐analytic evidence [19].

### 2.8. Certainty of Evidence Assessment

The certainty of evidence for the primary intervention and return‐to‐driving outcomes was assessed using the Grading of Recommendations Assessment, Development and Evaluation (GRADE) framework. Evidence certainty was evaluated according to the domains of RoB, inconsistency, indirectness, imprecision and publication bias. Outcomes were classified as high, moderate, low or very low certainty. Given the methodological heterogeneity and limited number of included studies, GRADE assessment was primarily applied to the major quantitative and clinically relevant outcomes synthesised in this review.

## 3. Results

### 3.1. Literature Search and Characteristics of the Included Studies

**Figure 1:**
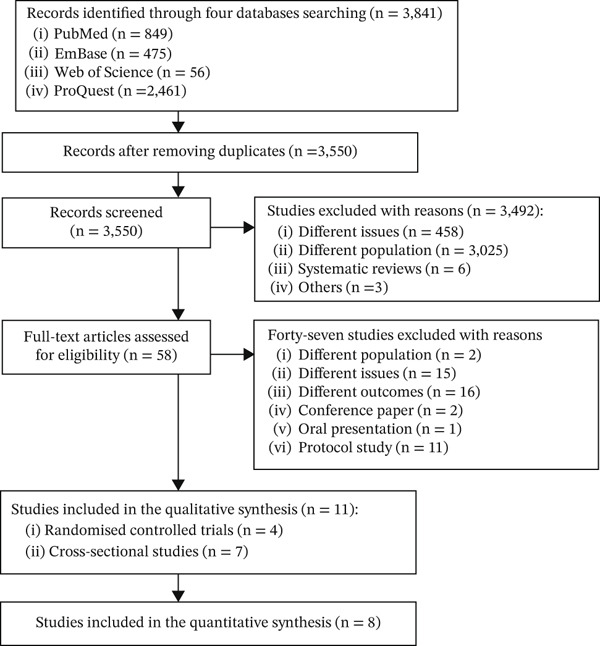
Preferred Reporting Items for Systematic Reviews and Meta‐Analyses (PRISMA) flow diagram of this review.

Based on the initial search strategies, we retrieved 3,841 records from four databases: PubMed (*n* = 849), Embase (*n* = 475), Web of Science (*n* = 56) and CINAHL (*n* = 2461). After excluding three duplicate studies, 3550 records were screened based on their titles and abstracts. Of these, 3492 records were excluded because they did not meet the inclusion criteria. The full texts of the remaining 58 articles were assessed for their eligibility. Forty‐seven studies were subsequently excluded because of disparate outcomes (*n* = 16), irrelevant topics (*n* = 15) and protocol studies (*n* = 11). Finally, 11 studies (four RCTs and seven cross‐sectional studies) were included in the qualitative synthesis [2, 3, 6, 8, 20–26]. Of these, eight studies provided sufficient data for quantitative synthesis, while the remaining studies were excluded from the meta‐analysis because of insufficient quantitative data (Figure 1).

### 3.2. Qualitative Assessment of the Observational Studies Included

The characteristics of the seven observational studies are summarised in Table 1. The study design was primarily cross‐sectional and retrospective, and 1681 participants were enrolled. The sample sizes varied widely across studies, ranging from 17 to 706 [2, 3, 23, 25]. However, all studies compared participants based on whether they returned to driving or passed a driving test. The Glasgow Coma Scale (GCS), which reflects the severity of the initial brain injury [8, 20, 23, 26]; functional independence measure (FIM), which indicates functional status; and post‐traumatic amnesia scale (PTA) are commonly reported as predictors of outcomes [2, 20, 23, 25, 26]. Specifically, for GCS scores, Ross et al. (2016) found that the return‐to‐drive group (or pass group) had significantly higher scores than the non‐return group [26], whereas Cullen et al. (2014) reported no significant difference between the two groups [23], demonstrating inconsistent results across studies. In reporting adherence using the STROBE checklist, six studies achieved high scores of 20 or higher, including Novack et al. (2021), with a perfect score of 22 [3], demonstrating excellent overall reporting (Table 1).

**Table 1:** Characteristics of observational studies of the association between acquired brain injury and automobile driving of the selected studies.

Author (year)	Aim	Study design, follow‐up period	Population (% female) Subgroup(age)	Outcome	Summary of results	Quality score
Cullen (2014)	To examine the utility of three common measures as predictors of return to driving after TBI	Cross‐sectional design	*N* = 200 (12%) *R* (*n* = 100, 49.77 ± 15.25) *NR* (*n* = 100, 51.42 ± 15.73)	GCS, FIM and DRS	*R* > *NR* on FIM *R* = *NR* on GCS and DRS	20/22
Labbe (2014)	To construct a parsimonious model predicting driving avoidance and exposure in light of demographic, cognitive and physical	Cross‐sectional study, prospective	*N* = 184 (29.9%)	Age, Sex, FIM motor, PTA, TMT, digit span and race	Older and female in driving avoidance PTA, TMT and digit span in driving exposure	20/22
Leon‐Carrion (2005)	To investigate whether post‐traumatic cognitive deficits prevent them from safely resuming driving	Cross‐sectional study, retrospective	*N* = 17 Drivers (*n* = 6) Nondrivers (*n* = 11)	FIM + FAM revised scale	Drivers > nondrivers on functionality index, especially sphincter control	13/22
Novack (2021)	To describe who can return to driving after moderate‐to‐severe	Cross‐sectional descriptive study	*N* = 706(27.9%) No attempt (*n* = 154, 44.9 ± 16.4) Driving stopped (*n* = 77, 44.1 ± 15.9) Driving now (*n* = 475, 45.7 ± 15.5)	TFC, PHQ‐9, SWLS, GOS‐E, PART‐O and driving survey	Driving now > No attempt = Driving stopped	22/22
Rapport (2008)	To examine resumption of driving after TBI and its relation to community integration	Cross‐sectional cohort study	*N* = 261(18.01%)	BDQ, CIM, CHART, driver survey and GCS	Drivers > nondrivers	21/22
Ross (2015)	To examine assessment outcomes and factors in on‐road driver assessment after TBI	Cross‐sectional study, retrospective	*N* = 207 *PG* (*n* = 137, 36.86 ± 14.69) *RG* (*n* = 36.76 ± 17.10)	GCS, PTA, RT, sex, physical impairment	*PG* > *RG* on GCS, PTA, RT, sex and physical impairment	21/22
Ross (2016)	To examine pre‐and post‐injury self‐reported driver behaviour and safety in returned drivers after TBI	Cross‐sectional study, Survey study	*N* = 106(24%) *PG* (*n* = 74, 43.40 ± 14.97) *RG* (*n* = 32, 44.12 ± 14.78)	Driving behaviour self‐reported issues	*PG* > *RG* on GCS and PTA	20/22
			Crashes reported (*n* = 30, 40.50 ± 15.75) No crashes reported (*n* = 76, 44.85 ± 14.39)	GCS, PTA duration, years licensed, sex and months driving since assessment	Crashes reported = No crashes reported	

Abbreviations: BDQ, barriers to driving questionnaire; CHART, Craig Hospital Assessment and Reporting Technique; CIM, community integration measure; DRS, disability rating scale; FAM, functional assessment measure; FIM, functional independence measure; GCS, Glasgow Coma Scale; GOS‐E, the Glasgow outcome scale extended; NR, non‐return to driving; PART‐O, participation assessment with recombined tools—objective scale; PG, pass group; PHQ‐9, Patient Health Questionnaire‐9; PTA, post‐traumatic amnesia scale; R, return to driving; RG, rehabilitation group; RT, reaction time; SWLS, satisfaction with life scale; TFC, time to follow commands; TMT, trail making test.

### 3.3. Qualitative Assessment of the Included RCT Studies

The detailed characteristics of the four RCTs are summarised in Table 2 [6, 21, 22, 24]. A total of 83 participants were included, with sample sizes ranging from 11 to 35 in each study. The experimental groups received driving simulators or VR‐based driving rehabilitation, whereas the control group received usual care or was placed on a waitlist for the intervention. The intensity and duration of the interventions varied, ranging from 45 to 90 min per session, with a total of four to 14 sessions each. The outcome measures included driving performance, cognitive function and driving‐related self‐efficacy. All four studies consistently reported that the experimental group demonstrated superior outcomes compared with the control group. Adherence to reporting using the CONSORT checklist showed a clear trend of improvement over time. Although the earlier study by Cox et al. (2010) received a relatively low score of 9 out of 25 [22], the recent studies by Bassingthwaighte et al. (2024) and Dimech‐Betancourt et al. (2021) achieved high scores of 22.5 and 21.5, respectively [21, 24], indicating significant advancements in the methodological quality and reporting of driving rehabilitation research (Table 2).

**Table 2:** Synthesising population, intervention, comparison and outcome in the selected randomised controlled trials studies.

Author (year)	No. of participants	Intervention	Provider/professional role	Therapeutic Intensity	Comparison	Progress monitoring	Summary of results	Quality score
Bassingthwaighte (2024)	*N* = 35 EG (*n* = 18, 52.78 ± 12.98) CG (*n* = 17, 46.18 ± 12.00)	Driving remediation programme + usual practice	OT‐led driving remediation in collaboration with driving rehabilitation specialists	60–90 min, 7–14 sessions	Usual practice	DASS‐21, DHQ, ADSES, MoCA, TMT and RPWT	EG > CG	22.5/25
Cox (2010)	*N* = 11 EG (*n* = 6, 26.2) CG (*n* = 5, 26.6)	VRDSRT and residential rehabilitation	Researcher‐delivered simulator‐based rehabilitation integrated with multidisciplinary residential rehabilitation (occupational, speech and psychotherapy services)	60–90 min, 4–6 sessions	Residential rehabilitation	RRQ and CARDS	EG > CG	9/25
Dimech‐Betancourt (2021)	*N* = 20 EG (*n* = 12, 53.92 ± 17.63) CG (*n* = 8, 41.63 ± 18.63)	Driving simulator intervention with usual care	OT driving rehabilitation team	45 min, 8 sessions	Usual care	ODP and SSQ	EG > CG on DCS‐D only	21.5/25
Ettenhofer (2019)	*N* = 17 EG (*n* = 11, 49.73 ± 9.05) CG (*n* = 6, 56.17 ± 7.13)	Neurocognitive driving rehabilitation	Examiner‐guided neurocognitive VR rehabilitation delivered by rehabilitation research staff under neuropsychological supervision	90 min, 6 sessions	Wait list	VR quotient, WAIS‐IV, TMT, LF, AF, CVLT‐II, GPD, PTSD checklist, BDI, SLS, SF‐36, ESS and FSS	EG > CG	17.5/25

Abbreviations: ABI, acquired brain injury; ADSES, Adelaide driving self‐efficacy scale; AF, animal fluency; AQPF, awareness questionnaire‐patient form; BDI, Beck Depression Inventory‐II total; BIDSAM, Brain Injury Driving Self‐Awareness Measure; CARDS, Cox Assessment of Risky Driving Scale; DASS‐21, Depression Anxiety Stress Scales—Short Form; DCS‐D, Driving Comfort Scale—Daytime; DHQ, driving history questionnaire; DOS, driver observation schedule; ESS, Epworth Sleepiness Scale; FSS, Fatigue Severity Scale; LF, letter fluency; MoCA, Montreal Cognitive Assessment; ODP, on‐road driving performance; RPWT, rapid pace walk test; RRQ, road rage questionnaire; SLS, satisfaction With Life Scale Total; SSQ, simulator sickness questionnaire; TBI, traumatic brain injury; TMT, trail making test Part A and Part B; VRDSRT, virtual reality driving simulation rehabilitation training; VR quotient, VR tactical driving and operational driving quotient.

### 3.4. Methodological Quality and RoB Assessments for the Observational Studies Included

The assessment results of the STROBE checklist and JBI appraisal tools for the seven included cross‐sectional studies are summarised in Tables 1 and 3, respectively. The assessment of adherence to the 23 STROBE items (Table 1) revealed that most studies adequately reported the title, abstract, background and discussion sections. However, few studies have explicitly stated the justification for the sample size calculation in the Methods section [2, 8, 23, 25, 26]. Additionally, in the results section, the handling of missing data or the flow of participants was not reported in some studies [2, 8, 25]. The overall reporting rate of the included studies was 80.

**Table 3:** Risk of bias of the selected observational studies based on JBI critical appraisal tool.

Risk of bias	Cullen (2014)	Labbe (2014)	Leon‐Carrion (2005)	Novack (2021)	Rapport (2008)	Ross (2015)	Ross (2016)
Inclusion criteria	Yes	Yes	Unclear	Yes	Yes	Yes	Yes
Subjects and setting	Yes	Yes	Yes	Yes	Yes	Yes	Yes
Exposure measurement	Yes	Yes	Yes	Yes	Yes	Yes	Yes
Condition measurement	Yes	Yes	Unclear	Yes	Yes	Yes	Unclear
Confounding identified	Yes	Yes	No	Yes	Yes	Yes	Yes
Confounding strategy	Yes	Yes	No	Yes	Yes	Yes	Unclear
Outcome measurement	Yes	Unclear	Yes	Yes	n/a	Yes	Unclear
Statistical analysis	Yes	Yes	Unclear	Yes	Yes	Yes	Yes

The methodological quality of the seven observational studies was evaluated using a modified JBI checklist consisting of eight items (Table 3). Four studies satisfied all the applicable criteria, indicating a low RoB [3, 8, 20, 23]. Leon‐Carrion et al. (2005) showed a high RoB because they failed to identify confounding factors or state strategies to address them [25]. Ross et al. (2016) reported unclear results regarding confounding management strategies [26]. Although most studies used valid and reliable outcome measures, the reliability of the outcome assessment remains unclear [2, 26]. Six studies used appropriate statistical methods, whereas Leon‐Carrion et al. (2005) did not [25].

### 3.5. Methodological Quality and RoB Assessments for the Included RCT Studies

**Figure 2:**
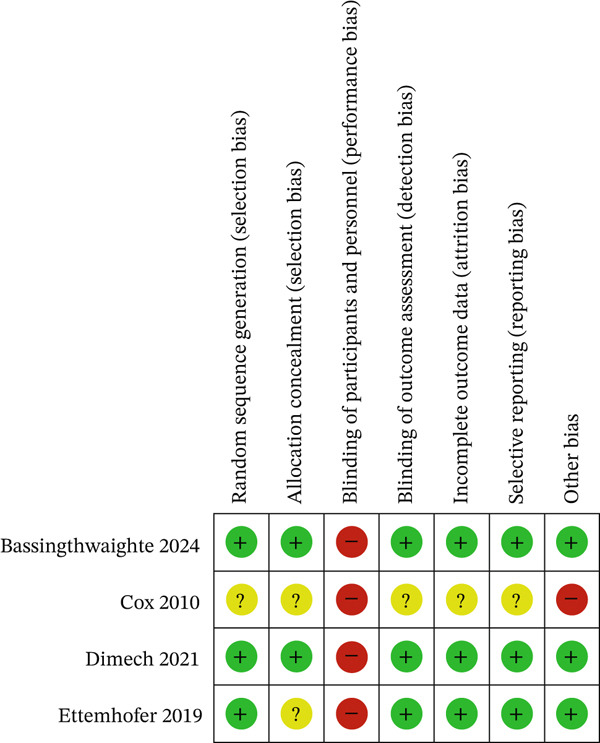
Risk of bias summary of this review.

The RCTs demonstrated a RoB across domains, including random sequence generation, allocation concealment and blinding of outcome assessment. A RoB summary is shown in Figure 2.

Performance bias (blinding of participants and personnel) was assessed as a high‐risk factor in all studies [6, 21, 22, 24]. This was anticipated because of the nature of rehabilitation interventions, in which blinding participants to active training is inherently challenging. However, selection bias (random sequence generation), detection bias (blinding of outcome assessment), attrition bias (incomplete outcome data) and reporting bias (selective reporting) were rated as low risk, suggesting that the outcome measures remained objective [6, 21, 24]. Cox et al. (2010) reported an unclear risk across several domains due to insufficient reporting of randomisation procedures, a lack of detailed reporting on sample size calculation and the specific implementation of randomisation procedures [22].

### 3.6. Quantitative Synthesis for the Included Observational Studies

**Figure 3:**
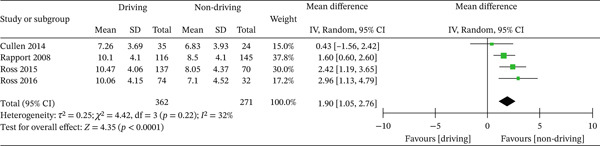
Glasgow coma scale score between driving and nondriving groups in observational studies.

Figure 3 presents a meta‐analysis comparing the initial injury severity measured using the GCS between the driving and nondriving groups. The analysis included four observational studies with 633 participants (362 in the driving group and 271 in the nondriving group) [8, 20, 23, 26]. The pooled analysis revealed that the driving group had significantly higher GCS scores than the nondriving group, with an MD of 1.90 (95% CI: 1.05–2.76, *p* < 0.0001). This indicates that individuals who successfully resumed driving had significantly less severe initial brain injuries. No significant heterogeneity was observed across the included studies (*I* ^2^ = 32%, *p* = 0.22), suggesting consistent results.

**Figure 4:**
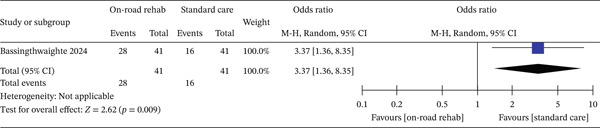
On‐road driving test pass rates for on‐road rehabilitation events and standard care events.

Figure 4 illustrates the effectiveness estimate from a single RCT evaluating the effectiveness of driving remediation on passing the on‐road driving assessment [21]. Of the 41 participants in each group, 28 (68.3%) in the rehabilitation group and 16 (39.0%) in the standard care group passed the test. Consequently, the intervention significantly increased the likelihood of passing the on‐road driving test, with an OR of 3.37 (95% CI: 1.36–8.35; *p* = 0.009).

**Figure 5:**
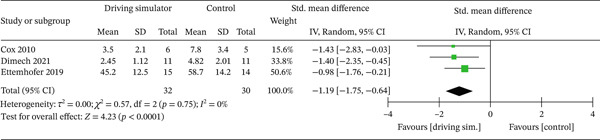
Driving errors between driving simulator and control groups in the RCT studies.

Figure 5 presents a meta‐analysis of driving errors, comparing the driving simulator and control groups. Three RCTs involving 62 participants were included in this analysis. The pooled results demonstrated a significant reduction in driving errors in the simulator group compared to the control group. The SMD was −1.19 (95% CI: −1.75 to −0.64; *p* < 0.0001), indicating a large effect size in favour of the intervention. Notably, no heterogeneity was observed across the included studies (*I* ^2^ = 0%, *p* = 0.75), suggesting that the effectiveness of simulator training in reducing driving errors was consistent across different research environments.

### 3.7. Certainty of Evidence Assessment

The certainty of evidence for the primary rehabilitation and return‐to‐driving outcomes was evaluated using the GRADE framework (Table 4). For simulator‐ and VR‐based rehabilitation interventions targeting driving errors, the certainty of evidence was rated as moderate. Although the pooled effect demonstrated a significant reduction in driving errors (SMD = −1.19, 95% CI: −1.75 to −0.64), the certainty was downgraded because of the relatively small sample size across the included RCTs. No serious concerns were identified regarding RoB, inconsistency or indirectness and heterogeneity was low (*I* ^2^ = 0%).

**Table 4:** GRADE certainty of evidence summary for rehabilitation effect and return‐to‐driving outcomes after acquired brain injury.

Outcome	Study design	No. of studies (participants)	Effect estimate	Risk of bias	Inconsistency	Indirectness	Imprecision	Publication bias	Overall certainty (GRADE)
Reduction in driving errors following simulator‐/VR‐based rehabilitation	RCTs	3 RCTs (*n* = 62)	SMD = −1.19 (95% CI: −1.75 to −0.64)	Not serious	Not serious	Not serious	Serious	Unable to assess ^∗^	MODERATE ⨁⨁⨁○
Passing on‐road driving assessment after rehabilitation	RCTs	1 RCT (*n* = 35)	OR = 3.37 (95% CI: 1.36–8.35)	Not serious	Not applicable	Not serious	Serious	Unable to assess	MODERATE ⨁⨁⨁○
Higher GCS scores predicting return to driving†	Observational study	4 studies (*n* = 633)	MD = 1.90(95% CI: 1.05‐2.76)	Not serious	Not serious	Not serious	Not serious	Unable to assess	LOW ⨁⨁○○

*Note:* GRADE working group grades of evidence: High certainty ⨁⨁⨁⨁: Very confident that the true effect lies close to the estimate. Moderate certainty ⨁⨁⨁○: Moderately confident in the effect estimate. Low certainty ⨁⨁○○: Limited confidence in the effect estimate. Very low certainty ⨁○○○: Very little confidence in the effect estimate.

Abbreviations: GCS, Glasgow Coma Scale; MD, mean difference; OR, odds ratio; RCT, randomised controlled trial; SMD, standardised mean difference; VR, virtual reality.

^∗^Publication bias could not be formally assessed because fewer than 10 studies were available for each pooled outcome, consistent with Cochrane recommendations.

†Calculated from studies providing extractable quantitative GCS data for pooled analysis.

For passing the on‐road driving assessment, the certainty of evidence was also rated as moderate. The included RCT demonstrated that participants receiving driving remediation were significantly more likely to pass the on‐road driving assessment than those receiving usual care (OR = 3.37, 95% CI: 1.36–8.35). However, the evidence was downgraded because the outcome was derived from a single study with a limited sample size.

The certainty of evidence for higher GCS scores as a predictor of return to driving was rated as low. Although the pooled analysis demonstrated a significant association between higher GCS scores and successful return to driving (MD = 1.90, 95% CI: 1.05–2.76), the evidence originated from observational studies. No additional downgrading was applied because heterogeneity was low (*I* ^2^ = 32%), the CI did not cross the null effect, and the outcome was directly relevant to return‐to‐driving status.

## 4. Discussion

This systematic review and meta‐analysis offers an updated assessment of the effectiveness of simulator‐based interventions and identifies key predictors of returning to driving in patients with ABI. It particularly focuses on the effects of simulator‐based training and the initial severity of the injury. The meta‐analysis found that simulator‐ and VR‐based training significantly reduced driving errors, showing a large effect size compared with the control conditions. The intervention increased the likelihood of passing the on‐road driving test by approximately 3.4 times. These results indicate that VR‐based training may provide a valuable approach for supporting the transition from clinical rehabilitation to return‐to‐driving outcomes. A prominent finding of this study was the lack of statistical heterogeneity across the included RCTs, indicating a high consistency in the treatment effect. The consistency of intervention effects across studies may reflect common therapeutic mechanisms related to repeated exposure and situational training. The observed consistency in treatment effects across studies aligns with the theory that repetitive exposure to hazardous virtual scenarios enhances cognitive processing speed and situational awareness, thereby improving driving performance. This is particularly relevant for occupation‐based training led by occupational therapists, where VR facilitates graded exposure and contextual adaptation to complex traffic situations, as demonstrated in recent studies [21, 24].

Although individual cross‐sectional studies have offered differing perspectives on the predictors of return to driving, such as Ross et al. (2015), who identified FIM as a significant determinant, our pooled meta‐analysis revealed that lower initial injury severity, indicated by higher GCS scores, was a significant predictor [20, 23]. These findings suggest that both historical injury severity and current functional status may be considered when evaluating return‐to‐driving readiness following ABI. Regular interventions could also benefit from incorporating VR‐based repetitive exposure to hazardous scenarios, enabling patients to develop coping skills safely. Additionally, the phenomenon of driving avoidance, as highlighted by Labbe (2014), underscores the importance of integrating psychological interventions with physical training to enhance driving self‐efficacy [2]. According to the literature, successfully completing a standard on‐road test does not necessarily ensure safe driving in real‐world conditions over the long term. For example, Ross et al. (2016) assessed actual driving metrics after individuals returned to driving and discovered that initial clinical indicators, such as the GCS and PTA, showed no significant differences between those who later reported crashes and those who did not [26]. This finding highlights potential limitations of relying solely on clinical evaluations or road test outcomes.

**Figure 6:**
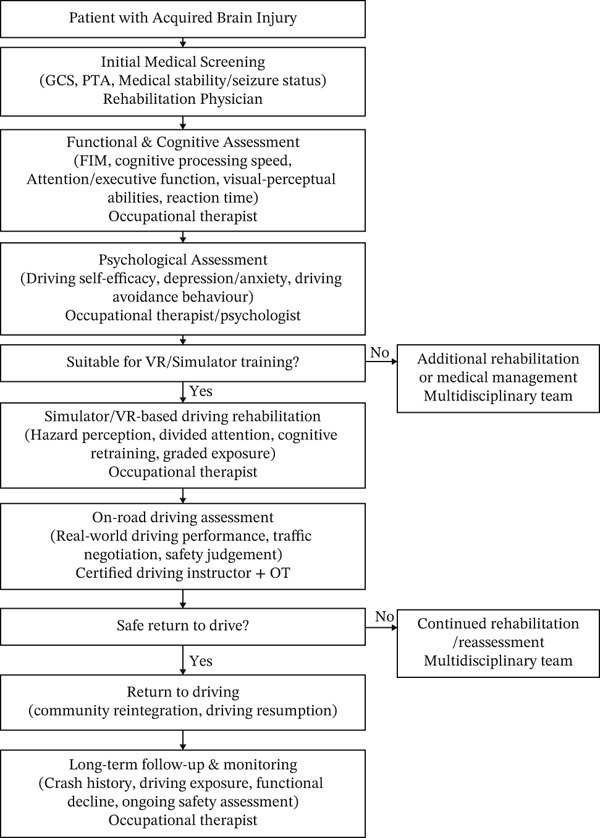
Proposed clinical decision‐making framework for return‐to‐driving rehabilitation after acquired brain injury. The framework integrates medical, cognitive‐functional, psychological, simulator‐based and on‐road assessments to support evidence‐based multidisciplinary driving rehabilitation and long‐term community reintegration. Abbreviations: FIM, functional independence measure; GCS, Glasgow coma scale; OT, occupational therapist; PTA, post‐traumatic amnesia.

This review has significant implications for establishing driving fitness standards in the era of partial automation [27, 28]. During the transition period before fully autonomous vehicles become commonplace, the ability to regain control during system limitations is crucial [27, 28]. As driving environments become more automated, future standards for driving rehabilitation and assessment may need to extend beyond basic vehicle operations [27, 29, 30]. Future research should explore how simulator‐based assessments of responsiveness to hazardous scenarios can inform safety guidelines or protocols during these transitional phases [30]. The observed decline in driving rehabilitation research, influenced by the transitional phase of autonomous driving technology and the ethical and financial constraints of on‐road assessments, limited the pool of available studies for our meta‐analysis, thereby restricting the breadth and depth of our conclusions [31–33]. This stagnation in driving rehabilitation research underscores the critical need for comprehensive syntheses, such as the present meta‐analysis. Consolidating evidence on existing interventions, such as VR and simulators, establishes a vital baseline for developing future rehabilitation protocols in the evolving landscape of autonomous driving [6, 15, 21, 34]. Importantly, intervention‐related findings derived from RCTs and predictive associations identified from observational studies should be interpreted separately because of differences in methodological design and levels of evidence. Figure 6 is the proposed clinical decision‐making framework for return‐to‐driving rehabilitation after acquired brain injury.

### 4.1. Implications for Evidence‐Based Occupational Therapy Practice

This review suggests a comprehensive, multidimensional strategy to guide evidence‐based occupational therapy. We propose a structured clinical framework encompassing four key domains [6, 22, 24, 26]. Initially, during the evaluation phase, occupational therapists must establish clear assessment pathways. Nonmodifiable screening predictors, such as initial injury severity (e.g., GCS), should be used to determine baseline rehabilitation eligibility. This must be complemented by assessing current functional independence (e.g., FIM) and physical, cognitive or visual recovery [8, 20, 23, 26]. Secondly, merely assessing physical, cognitive and visual recovery is insufficient; clinicians must proactively identify psychological barriers, such as depression and diminished self‐efficacy (e.g., the ADES), to prevent driving avoidance in patients who are otherwise functionally capable. [20, 21, 26]. Third, within these decision trees, occupational therapists must adeptly navigate the choice between simulators and on‐road training. Simulator‐ or VR‐based training should serve as a foundational, risk‐free platform for graded exposure to hazardous scenarios, effectively addressing driving avoidance and cognitive deficits. Once patients achieve sufficient mastery and self‐efficacy in the simulator, they can be safely transitioned to on‐road training, which offers the necessary ecological validity for real‐world applications. Finally, occupational therapists play a crucial role in long‐term follow‐up and monitoring. Passing a standard on‐road test is merely a milestone and does not guarantee lifelong safety. It is vital for occupational therapists to establish long‐term follow‐up plans to evaluate performance in real‐world settings by tracking actual driving metrics and post‐crash records. This ongoing commitment is essential to ensure sustained safety and achieve successful community‐based driving outcomes in the future.

The findings of this review can be effectively contextualised within fundamental occupational therapy frameworks. From the perspective of the person–environment–occupation–performance (PEOP) model, driving simulator and VR interventions serve as essential environmental adaptations. Driving is an intricate occupation that demands the simultaneous integration of motor and cognitive responses [6, 9, 10]. VR offers a controlled, risk‐free environment in which patients can repeatedly encounter complex and hazardous scenarios that are challenging to replicate on the road [21, 24]. This targeted person–environment interaction ultimately enhances occupational performance, as demonstrated by the substantial reduction in driving errors and the significantly increased likelihood of passing the on‐road test found in our meta‐analysis.

From the Model of Human Occupation (MOHO) perspective, resuming driving is not just about motor skills; it involves restoring a patient′s habituation, encompassing their daily routines and community roles [1–4]. Driving avoidance underscores the significance of the volitional subsystem. Even with sufficient performance capacity, a lack of driving self‐efficacy can impede the return to driving [2]. Consequently, occupational therapists employ simulator‐based training to address specific cognitive deficits and facilitate successful graded mastery experiences that rebuild patients′ volition and self‐efficacy for community reintegration.

### 4.2. Limitations

Throughout this review, there were no significant departures from the registered PROSPERO protocol. However, the review did reveal certain limitations. The relatively small number of studies included in each quantitative synthesis restricted both statistical power and generalizability. Consequently, the potential for publication bias and small study effects cannot be entirely ruled out, and conducting comprehensive sensitivity analyses was not possible. The reliability of the pooled estimates should be approached with caution. There was considerable variation in outcome measures and definitions across studies, especially concerning driving performance, self‐efficacy, cognitive function and return‐to‐driving outcomes. This diversity also hindered direct comparisons between studies and made standardised quantitative synthesis less feasible. Lastly, the review only considered studies published in English, which may have led to language bias and the omission of pertinent evidence available in other languages.

## Funding

This study was supported by Chosun University (10.13039/501100002457) (2024).

## Conflicts of Interest

The authors declare no conflict of interest.

## Data Availability

The data supporting the findings of this study are available from the corresponding author upon reasonable requests.

## References

[1] Christensen J. and Mcgrew C. A. , When Is It Safe to Drive after Mild Traumatic Brain Injury / Sports-related Concussion?, Current Sports Medicine Reports. (2019) 18, no. 1, 17–19, 10.1249/JSR.0000000000000558, 30624330. 30624330

[2] Labbe D. R. , Vance D. E. , Wadley V. , and Novack T. A. , Predictors of Driving Avoidance and Exposure Following Traumatic Brain Injury, Journal of Head Trauma Rehabilitation. (2014) 29, no. 2, 185–192, 10.1097/HTR.0b013e3182795211, 23474877. 23474877 PMC4487624

[3] Novack T. A. , Zhang Y. , Kennedy R. , Rapport L. J. , Watanabe T. K. , Monden K. R. , Dreer L. E. , Bergquist T. , Bombardier C. , Brunner R. , Goldin Y. , Marwitz J. , and Niemeier J. P. , Return to Driving After Moderate-to-Severe Traumatic Brain Injury: A Traumatic Brain Injury Model System Study, Archives of Physical Medicine and Rehabilitation. (2021) 102, no. 8, 1568–1575, 10.1016/j.apmr.2021.02.006. 33705772

[4] Fiedler A. M. , Almeida T. , Vasconcellos F. D. N. , Morell A. , de Monaco B. A. , Anghinah R. , and Cordeiro J. G. , Fitness-To-Drive After Adult Civilian Traumatic Brain Injury: Protocol for a Systematic Review and Meta-Analysis, Neurosurgical Review. (2023) 46, no. 1, 10.1007/s10143-023-02228-5, 38048009. 38048009

[5] Id I. K. , Vaucher P. , Cardoso I. , and Id B. F. , Assessment of Cognitive Screening Tests as Predictors of Driving Cessation: A Prospective Cohort Study of a Median 4-Year Follow-Up, PLoS One. (2021) 16, no. 8, e0256527, 10.1371/journal.pone.0256527, 34415967. 34415967 PMC8378690

[6] Ettenhofer M. L. , Guise B. , Brandler B. , Bittner K. , Gimbel S. I. , Cordero E. , Nelson Schmitt S. , Williams K. , Cox D. , Roy M. J. , and Chan L. , Neurocognitive Driving Rehabilitation in Virtual Environments (NeuroDRIVE): A Pilot Clinical Trial for Chronic Traumatic Brain Injury, NeuroRehabilitation. (2019) 44, no. 4, 531–544, 10.3233/NRE-192718, 31256093. 31256093 PMC6700618

[7] Imhoff S. and Lavalli M. , Driving Assessment and Rehabilitation Using a Driving Simulator in Individuals With Traumatic Brain Injury: A Scoping Review, NeuroRehabilitation. (2016) 39, no. 2, 239–251, 10.3233/NRE-161354, 27372359. 27372359

[8] Rapport L. J. , Bryer R. C. , Hanks R. A. , and Lj A. R. , Driving and Community Integration After Traumatic Brain Injury, Archives of Physical Medicine and Rehabilitation. (2008) 89, no. 5, 922–930, 10.1016/j.apmr.2008.01.009, 18452742. 18452742

[9] Mckerral M. , Moreno A. , Delhomme P. , and Gélinas I. , Driving Behaviors 2–3 Years After Traumatic Brain Injury Rehabilitation: A Multicenter Case-Control Study, Frontiers in Neurology. (2019) 10, 10.3389/fneur.2019.00144, 30899239. PMC6417438 30899239

[10] Wolfe P. L. and Lehockey K. A. , Neuropsychological Assessment of Driving Capacity, Archives of Clinical Neuropsychology. (2016) 31, no. 6, 517–529, 10.1093/arclin/acw050. 27474026

[11] Tran E. M. , Lee J. E. , and Requirements R. , Reporting Requirements, Confidentiality, and Legal Immunity for Physicians Who Report Medically Impaired Drivers, JAMA Network Open. (2024) 7, no. 1, e2350495, 10.1001/jamanetworkopen.2023.50495, 38180760. 38180760 PMC10770772

[12] Redelmeier D. A. , Yarnell C. J. , Thiruchelvam D. , and Tibshirani R. J. , Physicians′ Warnings for Unfit Drivers and the Risk of Trauma From Road Crashes, New England Journal of Medicine. (2012) 367, no. 13, 1228–1236, 10.1056/NEJMsa1114310, 23013074. 23013074

[13] Chi K. , Chen J. , Zhou S. , and Han Z. , The Effectiveness of Digital Cognitive Intervention in Patients With Traumatic Brain Injury: Systematic Review and Meta-Analysis, Frontiers in Neurology. (2025) 16, 1651443, 10.3389/fneur.2025.1651443, 41127285. 41127285 PMC12537419

[14] Chanmas G. , Taveekitworachai P. , Paliyawan P. , Thawonmas R. , Thawonmas R. , Nukoolkit C. , and Dajpratham P. , Driving Scenarios and Environmental Settings in Simulator-Based Driving Assessment Systems for Stroke: A Systematic Review, Topics in Stroke Rehabilitation. (2023) 30, no. 8, 872–880, 10.1080/10749357.2023.2165273, 36617424. 36617424

[15] Bassingthwaighte L. , Gustafsson L. , Griffin J. , and Fleming J. , Evaluating the Effectiveness of On-Road Driving Remediation Following Acquired Brain Injury: A Wait-List Feasibility Study With Follow-Up, Australian Occupational Therapy Journal. (2021) 68, no. 2, 124–134, 10.1111/1440-1630.12694. 32909309

[16] Cuschieri S. , The STROBE Guidelines, Saudi Journal of Anaesthesia. (2019) 13, no. Supplement 1, S31–S34, 10.4103/sja.SJA_543_18, 30930717. 30930717 PMC6398292

[17] Barker T. H. , Hasanoff S. , Aromataris E. , Stone J. C. , Leonardi-Bee J. , Sears K. , Habibi N. , Klugar M. , Tufanaru C. , Moola S. , and Liu X. L. , The Revised JBI Critical Appraisal Tool for the Assessment of Risk of Bias for Analytical Cross-Sectional Studies, JBI Evidence Synthesis. (2025) 23, no. 3, 441–453, 10.11124/JBIES-24-00523. 39177422

[18] Merkow R. P. , Kaji A. H. , and Itani K. M. F. , The CONSORT Framework, JAMA Surgery. (2021) 156, no. 9, 877–878, 10.1001/jamasurg.2021.0549, 33825818. 33825818 PMC8822473

[19] Higgins J. P. T. , Thomas J. , Chandler J. , Cumpston M. , Li T. , Page M. J. , and Welch V. A. , Cochrane Handbook for Systematic Reviews of Interventions, 2014, The Cochrane Collaboration.

[20] Ross P. E. , Ponsford J. L. , and Stefano D. , Predictors of On-Road Driver Performance Following Traumatic Brain Injury, Archives of Physical Medicine and Rehabilitation. (2015) 96, no. 3, 440–446, 10.1016/j.apmr.2014.09.027, 25316183. 25316183

[21] Bassingthwaighte L. , Gustafsson L. , Molineux M. , Bell R. , Perez W. , and Shah D. , On-Road Driving Remediation Following Acquired Brain Injury: A Randomized Controlled Trial, Brain Injury. (2024) 38, no. 13, 1113–1124, 10.1080/02699052.2024.2376763, 38994668. 38994668

[22] Cox D. J. , Davis M. , Singh H. , Barbour B. , Don Nidiffer F. , Trudel T. , Mourant R. , and Moncrief R. , Driving Rehabilitation for Military Personnel Recovering From Traumatic Brain Injury Using Virtual Reality Driving Simulation: A Feasibility Study, Military Medicine. (2010) 175, no. 6, 411–416, 10.7205/MILMED-D-09-00081, 20572473. 20572473

[23] Cullen N. , Krakowski A. , and Taggart C. , Functional Independence Measure at Rehabilitation Admission as a Predictor of Return to Driving After Traumatic Brain Injury, Brain Injury. (2014) 28, no. 2, 189–195, 10.3109/02699052.2013.862738, 24456058. 24456058

[24] Dimech-Betancourt B. , Ponsford J. L. , Charlton J. L. , Ross P. E. , Gooden J. R. , and Stolwyk R. J. , Investigating Feasibility and Preliminary Efficacy of a Simulator- Based Driving Intervention for People With Acquired Brain Injury: A Randomised Controlled Pilot Study, Clinical Rehabilitation. (2021) 35, no. 9, 1277–1289, 10.1177/02692155211002455, 33810776. 33810776

[25] Leon-Carrion J. , Dominguez-Morales M. , and Martin J. B. Y. , Driving With Cognitive Deficits: Neurorehabilitation and Legal Measures Are Needed for Driving Again After Severe Traumatic Brain Injury, Brain Injury. (2005) 19, no. 3, 213–219, 10.1080/02699050400017205, 15832895. 15832895

[26] Ross P. , Ponsford J. L. , Di Stefano M. , Charlton J. , and Spitz G. , On the Road Again After Traumatic Brain Injury: Driver Safety and Behaviour Following On-Road Assessment and Rehabilitation, Disability and Rehabilitation. (2016) 38, no. 10, 994–1005, 10.3109/09638288.2015.1074293, 26312651. 26312651

[27] Chen H. , Zhao X. , Chen C. , Li Z. , Li H. , and Wang Q. , A Systematic Review on Test Performance of the Driver Takeover Process in Automated Driving, Accident Analysis and Prevention. (2025) 215, 108012, 10.1016/j.aap.2025.108012. 40121970

[28] Weaver B. W. and DeLucia P. R. , A Systematic Review and Meta-Analysis of Takeover Performance During Conditionally Automated Driving, Human Factors. (2022) 64, no. 7, 1227–1260, 10.1177/0018720820976476, 33307821. 33307821

[29] Classen S. , Gelinas I. , Barco P. , Gibson B. , Haffner E. , Jeghers M. , Wandenkolk I. , and Devos H. , Automated Vehicles: Future Initiatives for Occupational Therapy Practitioners and Driver Rehabilitation Specialists, Occupational Therapy Journal of Research. (2024) 44, no. 4, 543–553, 10.1177/15394492241229993, 38389336. 38389336

[30] Sever T. and Contissa G. , Automated Driving Regulations—Where Are We Now?, Transportation Research Interdisciplinary Perspectives. (2024) 24, 101033, 10.1016/j.trip.2024.101033.

[31] Lajunen T. and Sullman M. J. M. , Attitudes Toward Four Levels of Self-Driving Technology Among Elderly Drivers, Frontiers in Psychology. (2021) 12, 682973, 10.3389/fpsyg.2021.682973, 34248785. 34248785 PMC8261150

[32] Classen S. , Sisiopiku V. P. , Mason J. R. , Yang W. , Hwangbo S. W. , McKinney B. , and Li Y. , Experience of Drivers of All Age Groups in Accepting Autonomous Vehicle Technology, Journal of Intelligent Transportation Systems. (2024) 28, no. 5, 651–667, 10.1080/15472450.2023.2197115.

[33] Bellagamba D. , Vionnet L. , Margot-Cattin I. , and Vaucher P. , Standardized On-Road Tests Assessing Fitness-To-Drive in People With Cognitive Impairments: A Systematic Review, PLoS One. (2020) 15, no. 5, e0233125, 10.1371/journal.pone.0233125, 32421733. 32421733 PMC7233547

[34] Palubiski L. and Crizzle A. M. , Evidence Based Review of Fitness-To-Drive and Return-To-Driving Following Traumatic Brain Injury, Geriatrics. (2016) 1, no. 3, 10.3390/geriatrics1030017. PMC6371138 31022811

